# A classical PKA inhibitor increases the oncolytic effect of M1 virus via activation of exchange protein directly activated by cAMP 1

**DOI:** 10.18632/oncotarget.10305

**Published:** 2016-06-27

**Authors:** Kai Li, Jiankai Liang, Yuan Lin, Haipeng Zhang, Xiao Xiao, Yaqian Tan, Jing Cai, Wenbo Zhu, Fan Xing, Jun Hu, Guangmei Yan

**Affiliations:** ^1^ Department of Pharmacology, Zhongshan School of Medicine, Sun Yat-sen University, Guangzhou 510080, China; ^2^ Department of Microbiology, Zhongshan School of Medicine, Sun Yat-sen University, Guangzhou 510080, China; ^3^ Collaborative Innovation Center for Cancer Medicine, Sun Yat-sen University, Guangzhou 510080, China; ^4^ Department of Pharmacy, The Third Affiliated Hospital of Sun Yat-sen University, Guangzhou 510080, China

**Keywords:** oncolytic virus, M1 virus, Epac1, H89, innate immunity

## Abstract

Oncolytic virotherapy is an emerging and promising treatment modality that uses replicating viruses as selective antitumor agents. Here, we report that a classical protein kinase A (PKA) inhibitor, H89, synergizes with oncolytic virus M1 in various cancer cells through activation of Epac1 (exchange protein directly activated by cAMP 1). H89 substantially increases viral replication in refractory cancer cells, leading to unresolvable Endoplasmic Reticulum stress, and cell apoptosis. Microarray analysis indicates that H89 blunts antiviral response in refractory cancer cells through retarding the nuclear translocation of NF-κB. Importantly, *in vivo* studies show significant antitumor effects during M1/H89 combination treatment. Overall, this study reveals a previously unappreciated role for H89 and demonstrates that activation of the Epac1 activity can improve the responsiveness of biotherapeutic agents for cancer.

## INTRODUCTION

Oncolytic viruses (OVs) are therapeutically useful viruses that selectively infect and damage cancerous tissues without causing harm to normal tissues [1, 2]. An increasing number of viruses including measles virus, adenovirus, poxvirus, vesicular stomatitis virus, and herpes simplex virus can be adapted to cancer therapies for their restricted replication in cancer cells before or after engineering and some of them have advanced deeply into clinical trials [3–9]. Of note, Talimogene laherparepvec (T-VEC), a herpes simplex virus type 1–derived oncolytic immunotherapy designed to selectively replicate within tumors and produce granulocyte macrophage colony-stimulating factor (GM-CSF) to enhance antitumor immune response, is the first approved oncolytic immunotherapy by the US Food and Drug Administration (FDA) demonstrated therapeutic benefit against melanoma [10–12].

We previously have identified that alphavirus M1 as a potential and promising antitumor agent that selectively targets various cancer cells *in vitro*, *in vivo* and *ex vivo* [13, 14]. M1 is an alphavirus isolated in the 1960s from the Hainan province of China, and belongs to the Togavirus family of viruses [15]. The genome of M1 is 11,690 nucleotides (nt) in length and contains two open reading frames, encoding four nonstructural proteins (nsP1-nsP2-nsP3-nsP4), and five structural proteins (C-E3-E2-6K-E1) [15]. What's more, the direct cell lytic effect of M1 is through prolonged and severe Endoplasmic reticulum(ER) stress induced apoptosis in cancer cells. Two central apoptotic pathways are activated: the Jun N-terminal kinase (JNK) pathway, and the Caspase-12 pathway, but not another C/EBP-homologous protein (CHOP) pathway [13].

Although oncolytic viruses inhibiting cancer cell growth *in vitro* is definitive, the antitumor effects of OVs can be limited by various cellular processes. For instance, intratumoral antiviral response plays a crucial role in blocking the therapeutic spread of oncolytic viruses [16]. Antiviral response is initiated in infected cells after detection of viral RNA by Pattern Recognition Receptors (PRRs) [17]. PRRs induce signaling cascades that activate latent transcription factors, including IFN regulatory factors (IRFs) and NF-κB. Activation of these genes lead to expression of virus responsive genes, including type I IFNs (IFN-α/β) and subsequently hundreds of different IFN-stimulated effector genes (ISGs) [18, 19]. Recently, microtubule destabilizing agents had also been found to lead to superior viral spread in cancer cells by disrupting type I IFN mRNA transcription, leading to decreased IFN protein expression and secretion [20].

Activation of cyclic adenosine monophosphate (cAMP) signal pathway has been reported to inhibit the innate immune response, lipopolysaccharide (LPS)- or polyinosinic:polycytidylic acid (Poly[I:C])-induced IFNs production [21–23]. The main identified downstream effector of cAMP includes PKA/CREB pathway, exchange protein directly activated by cAMP (Epac), and Cyclic nucleotide-gated (CNG) channels [24, 25]. In eukaryotic cells, cAMP/PKA/CREB pathway controls many cellular mechanisms such as gene transcription, ion transport, and protein phosphorylation [26]. Epac is a newly identified cAMP intracellular receptor, which has been implicated in regulating exocytosis and secretion, cell adhesion, endothelial barrier junctions and leptin signaling [27–30]. We can activate cAMP pathway through the adenylate cyclase activator Forskolin and the cellular permeable cAMP analogue db-cAMP. PKA inhibitor H89 has been used extensively for evaluation of the role of PKA and ESI-09 is a newly identified Epac1 specific inhibitor [31, 32].

During the study of the role of PKA, we accidentally find that PKA inhibitor H89 dramatically enhances the oncolytic effects of M1. In this study, we sought to investigate the anticancer effectiveness of M1/H89 combination treatment and uncover the mechanisms. Surprisingly, the underlying mechanism is due to activation of Epac1 guanine nucleotide exchanging activities and inhibition of p65 nucleus translocation. This study suggests that H89 has the potential to extensively enhance the spectrum of malignancies amenable to oncolytic virotherapeutics and indicates that Epac1 pathway is critical for oncolytic virotherapy.

## RESULTS

### Determination of oncolytic effects of M1 virus after PKA modulators treatments

**Figure 1:**
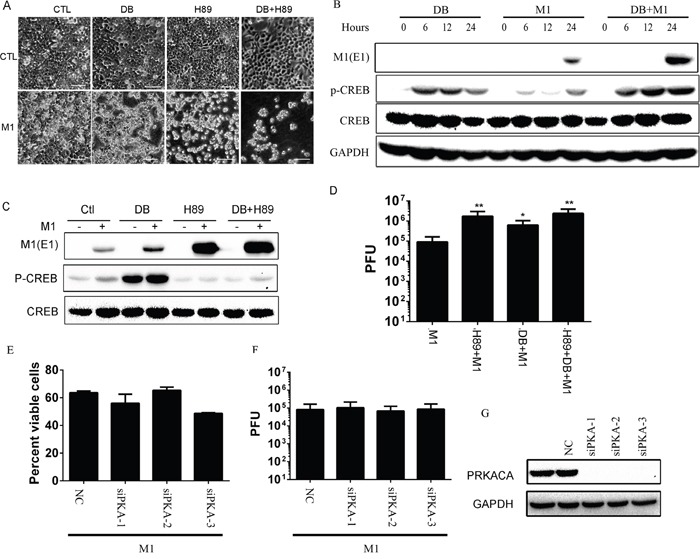
The oncolytic effects of M1 virus after PKA modulators treatments **A.** Morphological observation of HCT-116 after various treatments. Cells were pretreated with H89 (10μM) for 1 hour or not and then treated with db-cAMP (500μM) or M1 (1 PFU/cell). Pictures were captured with light microscope 72 hours post infection. CTL, control; DB, db-cAMP. Scale bars=50μm. **B.** db-cAMP treatment activates the PKA/CREB pathway. HCT-116 cancer cells were treated with db-cAMP (1 mM) or not in the presence or absence of M1 (1 PFU/cell) infection. **C.** H89 blocks the phosphorylated CREB and increases viral protein E1 expression. HCT-116 cancer cells were pretreated with H89 (10μM) for 1 hour or not and then treated with db-cAMP (500μM) or M1 (1 PFU/cell). Protein expressions were determined 24 hous post infection. **D.** H89 and db-cAMP increases the replication of M1 (mean ± SD). HCT-116 cancer cells were pretreated with H89 (10μM) for 1 hour and then treated with db-cAMP (500μM) or M1 (1 PFU/cell). Supernatant were collected 24 hous post infection and viral titers were determined. **E-G.** Knockdown of PKA does not increase the oncolytic effects of M1 virus. PKA catalytic subunit siRNA and scramble siRNA were transfected in HCT-116 cells and PKA expression was determined by westen blot 48 hour later (right). M1virus (1 PFU/cell) was infected after knockdown of PKA for 48 hours. Cell viabilities were determined 72 hours post infection and viral titers were determined 24 hours post infection. PRKACA, PKA catalytic subunit; *p<0.05; **p<0.01.

Previous findings from our laboratory have identified that activation of cAMP pathway increases the oncolytic activities of M1 [33]. During the exploration of the role of PKA, we chose the extensively used H89 to inhibit the kinase activities. With light microscope observation, irrespective of PKA activator db-cAMP, we find that PKA inhibitor H89 increases M1 induced cytopathic effects in colorectal cancer cell line HCT-116 (Figure 1A).

Furthermore, we observed that db-cAMP treatment activates PKA pathway through detection of phosphorylated CREB and H89 almost totally blocks the phosphorylation of CREB (Figure 1B and 1C). We next find that H89 increases viral protein expression (Figure 1C) and viral replication (Figure 1D). We performed RNAi to knockdown catalytic subunit of PKA. We found that silencing of PKA does not enhance the oncolytic effects and viral replication of M1(Figure 1E and 1F). The knockdown efficiency of PKA catalytic subunit is indicated in Figure 1G with immunoblot. These results demonstrate that the enhanced oncolytic effect of H89 is irrelevant to PKA and drive us to elucidate the underlying mechanisms, and subsequently, explore the antitumor effects of H89/M1 virus treatment.

### H89 enhances the oncolytic effects of M1 in various cancer cells

**Figure 2:**
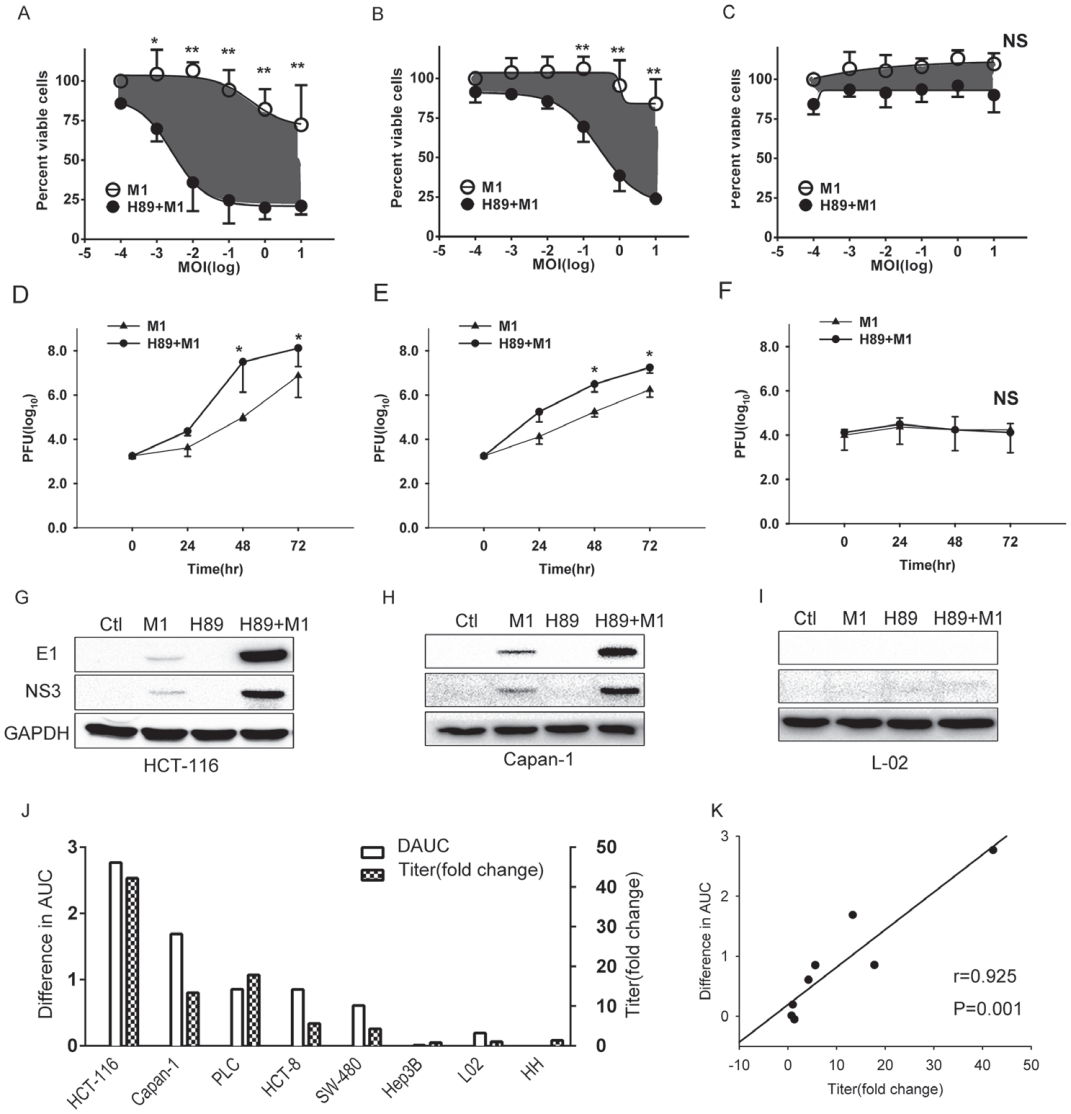
H89 selectively increases M1 replication and oncolysis in cancer cells **A-C.** Determination of cell viabilities with MTT assay. HCT-116, Capan-1 and L-02 cells were pretreated with H89 (10μM) for 1 hour, and then M1 virus was infected at 0, 0.001, 0.01, 0.1, 1, 10 PFU/cell. Cell viabilities were determined 72 hours postinfection with MTT assay. **D-F.** Viral titer determination in different cell lines. HCT-116, Capan-1 and L-02 cells were pretreated with H89 (10μM) and viral titers (supernatant) were determined at the 0, 24, 48, 72 hours postinfection (0.1PFU/cell) with TCID50. **G-I.** Western blots showing viral proteins E1 and NS3 expression 24 hours postinfection. Ctl, Control. **J.** Bar graphs depict the relative differences in AUC (area under the curve) (i.e., gray areas shown in A and B) and viral titer fold changes. H89(10μM) was treated and viral titers were determined 48 hours post infection (0.1PFU/cell). **K.** Correlation analysis of difference in AUC and fold change of viral titer. *p<0.05;**p<0.01; NS, not significant.

To determine the effect of H89 during oncolytic virus M1 infection, human cancer cell lines HCT-116 and Capan-1 and human immortalized cell line L-02 were pretreated with or without H89, and then infected with M1 virus at different plaque forming unit (PFU)/Cell. We found that H89 dramatically increases the oncolytic effects of M1 in cancer cells (Figure 2A and 2B), while no enhanced effects was observed in human immortalized liver cell line L-02 (Figure 2C).

Viral replication and spread is critical for oncolytic virotherapy. Thus, we next determined the viral titer and viral structural protein E1 and non-structural protein NS3 expression after H89 treatment. Our results show that H89 dramatically increases the viral replication in cancer cells in a time dependent manner and, importantly, it does not increase viral replication in normal cells (Figure 2D-2I).

To validate the antitumor effect, we detected the cell viabilities and viral titers of 6 cancer cells and 2 normal cells after treated with H89/M1. We found that H89 dramatically enhances the oncolytic effects of M1 and increases the replication of M1 in cancer cells (Figure 2J). Moreover, we observed a positive correlation between the enhanced oncolytic effects and increased viral replication (Figure 2K), which indicates that the enhanced oncolytic effects is due to increased viral replication.

### Enhanced oncolysis is due to prolonged and severe ER stress

Increased viral replication means accumulation of large amounts of viral proteins in ER lumen. ER transmembrane receptors detect the viral proteins and initiate the unfolded protein response (UPR) to restore normal ER function. If the adaptive response fails, apoptotic cell death ensues [34]. We hypothesized that the accumulation of viral proteins might induce ER stress, which leads to catastrophic destruction of ER and cellular apoptosis.

**Figure 3:**
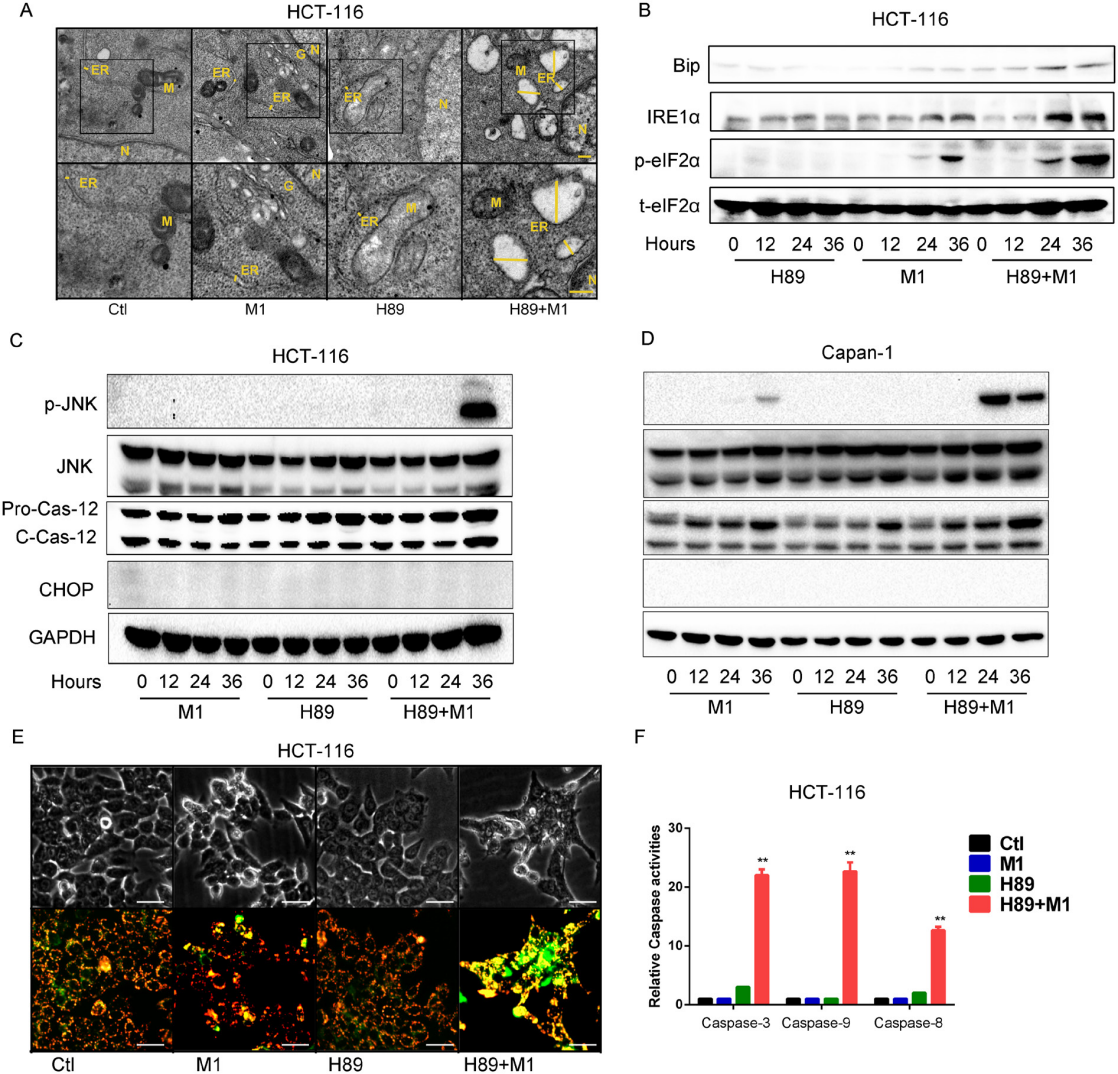
Enhanced oncolysis is due to irreversible ER stress **A.** Ultrastructural observation of HCT-116 cell line after H89/M1 treatment. The marker indicates the relative size of ER. Transmission electron microscopy (TEM) images (9,700, up; 13,800, down) of HCT-116 cells after 48hr of treatment with vehicle, M1 virus, H89, H89+M1; the marker indicates the relative size of the ER. N, nucleus; M, mitochondrion; G, Golgi apparatus; scale bars=250 nm. **B.** H89/M1 treatment induces ER stress marker expression. HCT-116 cells were pretreated with H89 (10μM) for 1 hours and subsequently infected with oncolytic virus M1 (1PFU/cell). Protein expressions were determined 0, 12, 24, 36 hours post infection. **C** and **D.** H89/M1 treatment induces phosphorylated JNK and cleaved-Caspase-12 expression. HCT-116 and Capan-1 cells were pretreated with H89 (10μM) for 1 hours and subsequently infected with oncolytic virus M1 (1PFU/cell). **E.** Mitochondrial potential staining with JC-1. Cells were pretreated with H89 (10μM) for 1 hour and then infected with M1 virus for 48 hours. **F.** Caspase-3, Caspase-9 and Caspase-8 activity assays (mean ± SD). HCT-116 cells were plated on 96-well plates and M1 virus was infected for 72 hours in the presence or absence of H89. GAPDH, glyceraldehyde-3-phosphate dehydrogenase. **P < 0.01.

To elucidate the biological consequences of H89/M1 combination treatment, through transmission electron microscope observation, we find that M1/H89 treatment induces striking swelling of ER in HCT-116 cancer cell line (Figure 3A). With ER stress marker detection, we further find that M1/H89 combination treatment increases Bip, IRE1α and phosphorylated eIF2α expression in HCT-116 cancer cells(Figure 3B).

Thus, we next sought to investigate the commonly known ER stress induced apoptotic pathways, including JNK, CHOP and Caspase-12 pathway. In HCT-116 and Capan-1 cancer cell lines, either H89 or M1 treatment alone does not activate the three pathways. While, H89/M1 combination treatment significantly activates phosphorylated JNK and slightly unregulated cleaved-Caspase12 (Figure 3C, 3D). Interestingly, CHOP was not increased after the single or combination treatment, indicating that this pathway might not be involved (Figure 3C, 3D).

Given that unresolvable ER stress usually leads to cell apoptosis, we next detected mitochondrial membrane potential and found that M1/H89 combination treatment induces mitochondrial potential loss, indicating that mitochondrial apoptotic pathway is activated(Figure 3E). We next detected the caspase activities in HCT-116 cancer cell line. Consistently, we find that H89/M1 combination treatment significantly activats the activities of Caspase-3/7, Caspase-9 and also Caspase-8 (Figure 3F), suggesting that downstream apoptotic pathways are activated. These data demonstrate that the combination treatment induced cell death is through ER stress induced apoptosis.

### Microarray analysis shows that H89 inhibits the innate antiviral response

**Figure 4:**
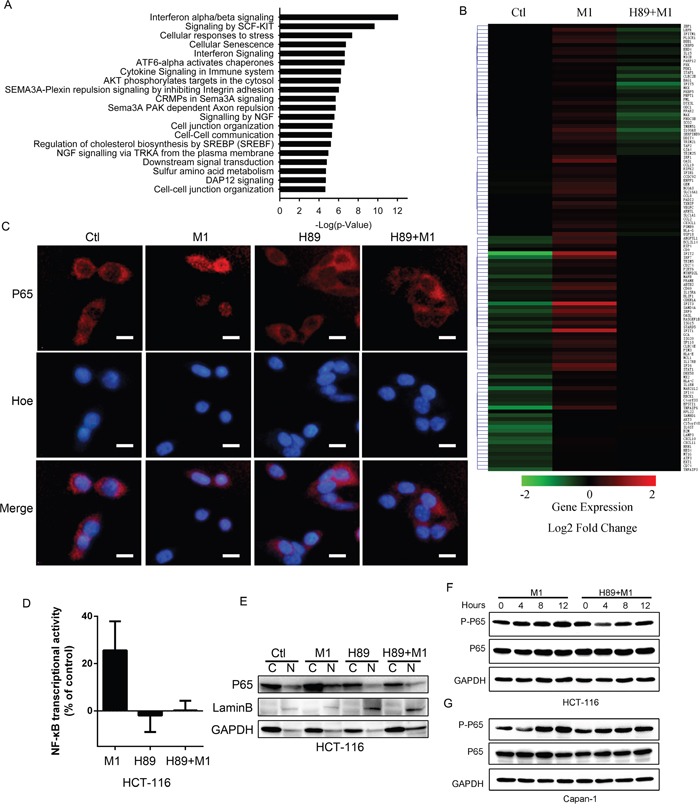
H89 inhibits type I IFN signaling pathway **A.** Gene Ontology analysis of the pathways overrepresented among genes differentially expressed between HCT-116 stimulated with vehicle or with M1. **B.** Heatmap of the expression of ISGs induced by M1 virus infection. **C.** Immunofluorescent staining of p65. HCT-116 cancer cells were pretreated with H89 for one hour and then infected with M1 virus (1PFU/cell) for four hours. **D.** NF-κB transcriptional activity determination. HCT-116 cells co-transfected with plasmids of pNF-κB-luciferase plus pRL-TK renilla and analyzed by luciferase reporter activity assays. **E.** Subcellular fractions of NF-B in cancer cells. Cytoplasmic and nuclear fractions were analyzed by IB analyses. Nuclear protein laminB was used as a nuclear (N) protein marker and GAPDH was used as a cytoplasmic (C) protein marker. **F** and **G.** Expression of phosphorylated p65 with western blot. HCT-116 and Capan-1 cancer cells were pretreated with H89 (10μM) or not and then infected with M1 virus (1 PFU/cell). Protein expressions were determined 0, 4, 8, 12 hours postinfection.

We have demonstrated that H89 enhances the oncolytic effects of M1 in various cancer cells via PKA-independent manner. We next sought to determine the underlying mechanisms. To determine how H89 affects gene expression to increase viral replication, we prepared HCT-116 cells mRNA to perform Affymetrix arrays. Gene ontology analysis of the differentially expressed genes between cells treated with vehicle or M1 revealed a highly significant enrichment of genes related to Interferon-α/β signaling pathway (Figure 4A).

Invading viruses will be recognized by host cells and induce interferon expression through activation of NF-κB [35]. Interferon induces diverse range of gene expressions to restrict virus replication and spread [18]. We compiled 329 ISGs and observed that H89 treatment abrogates the transcriptional upregulated ISGs by M1, demonstrating the inhibiting role of H89 on type I Interferon response (Figure 4B).

To validate the induced antiviral response is blocked by H89, we determined the interferon-inducing pathway. From Figure 4C-4E, we found that H89 treatment abrogates the nucleus translocation of p65 and inhibits NF-κB transcriptional activity post virus infection. With phosphorylated p65 detection, we validated that H89 abrogates M1 induced phosphorylation of p65 in HCT-116 and Capan-1 cancer cell lines (Figure 4F and 4G). These results demonstrate the critical involvement of NF-κB in H89 mediated type I IFN response inhibition.

### H89 enhances the oncolytic effects via activation of Epac1

**Figure 5:**
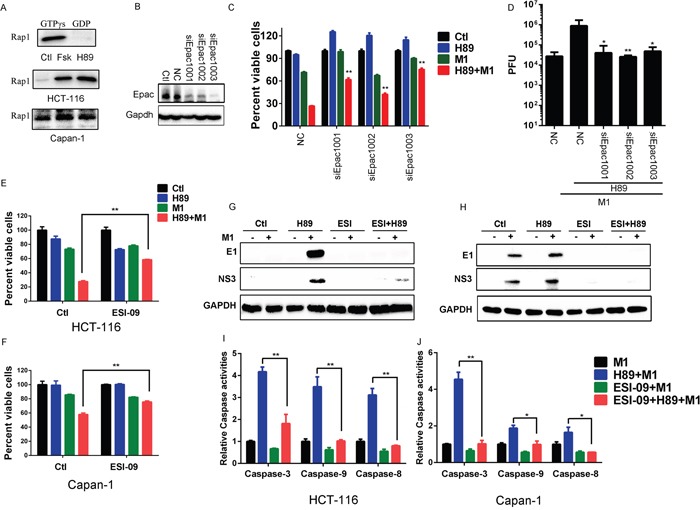
H89 activates Epac1 to increase oncolytic effects of M1 **A.** Activated Rap1 determination. HCT-116 and Capan-1 cancer cells were treated with Forskolin (10μM) or H89 (10μM) for 24 hours and active Rap1 was detected with the Active Rap1 Detection Kit. GTPγs and GDP indicates positive control and negative control, respectively. **B-D.** The effects of Epac1. HCT-116 cancer cells were transfected with small interfering RNA (siRNAs) against Epac1. Epac1 expression levels were determined (B). Cell viabilities (72 hours post infection) and viral titers (48 hours post infection) were determined in the presence or absence of H89 after siRNA transfection (C and D). **E** and **F.** Cell viabilities determination with MTT assay after different treatment. HCT-116 and Capan-1 cancer cells were pretreated with ESI-09 or H89 or both for 1 hour and then infected with M1 (0.1 PFU/cell). Cell viabilities were determined 72 hours post infection. **G** and **H.** Determination of viral proteins E1 and NS3 with western blot. HCT-116 and Capan-1 cells were pretreated with ESI-09 or H89 or both for 1 hour, then M1 virus (1PFU/cell) was infected. Protein expression was determined 24 hours postinfection. **I** and **J.** Caspase-3, Caspase-9 and Caspase-8 activity assays (mean ± SD). HCT-116 and Capan-1 cells were plated on 96-well plates and M1 virus was infected for 72 hours in the presence or absence of H89 and/or ESI-09. *p<0.05; **p< 0.01. GAPDH, glyceraldehyde-3-phosphate dehydrogenase.

Previous findings from our laboratory have identified that Epac1 activation increases the oncolytic activities of M1 through inhibition of the induced antiviral response. Hence, we reasoned if H89 affects the acitivity of Epac1. Epac1 is a guanine nucleotide exchanging factors that activates Rap1. With active Rap1 assay, we found that both H89 and Forskolin can upregulate GTP-Rap1 (Figure 5A). This result strongly suggests that the enhanced oncolysis is through Epac1 activation.

To validate that enhanced oncolysis is through Epac1, we performed RNA interference and found that, compared with scramble RNA group, knockdown of Epac1 significantly abrogate the enhanced oncolytic activity by H89 (Figure 5B and 5C). With viral titer determination, we also observed that knockdown of Epac1 cancels the increased viral replication by H89 (Figure 5D). We next used ESI-09 to block the activity of Epac1. In HCT-116 and Capan-1 cancer cells, we observed that ESI-09 significantly abolishes the enhanced oncolysis by H89, further suggesting that the enhanced oncolysis by H89 is through Epac1 (Figure 5E and 5F). Moreover, with ESI-09, we observed that inhibition of Epac1 abolishes the increased viral protein expression by H89 in HCT-116 and Capan-1 cancer cells (Figure 5G and 5H).

We have observed that the increased oncolytic activity is through ER stress induced apoptosis. Therefore, we detected the Caspase activities after inhibition of Epac1 with ESI-09. From Figure 5I and 5J, we found that ESI-09 abrogates the activities of Caspase-3/9/8 induced by H89/M1 combination treatment.

### H89/M1 combination treatment inhibits tumor growth *in vivo*

**Figure 6:**
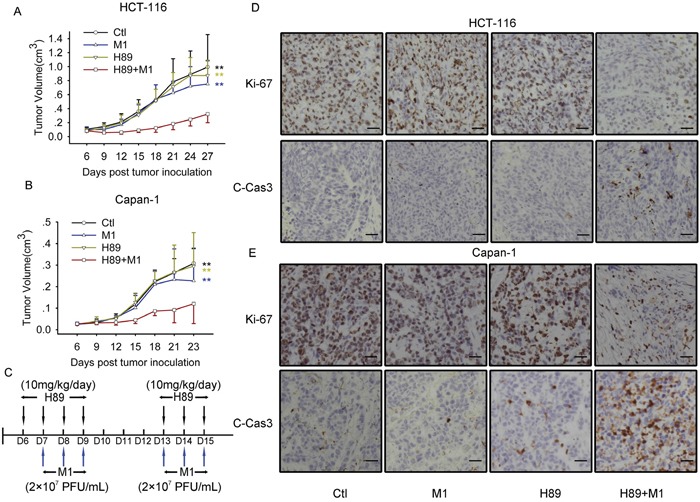
M1 plus H89 significantly reduces tumor size **A** and **B.** Measurement of the antitumor efficacy of M1 and/or H89 *in vivo*. Nude mice (NU/NU) bearing subcutaneous HCT-116 (A) and Capan-1 (B) tumors were treated with vehicle, H89 (10 mg/kg/day), M1 virus (2 × 10^7^ PFU/day), M1 virus and H89 (n ≥ 7). Tumor growth was assessed by tumor volume measurement over time (mean ± SD). At experimental endpoints, mice were anesthetized and sacrificed. i.v., intravenously injection (tail vein); **P < 0.01, compared with the combination group. **C.** Treatment schedule for HCT-116 and Capan-1 tumor model. **D** and **E.** Protein expressions in tumor mass with Immunohistochemistry staining. Immunohistochemistry was performed to analyze the expression of Ki-67 and Cleaved-Caspase-3. Scale bars=50μM.

Given the validated oncolytic efficacy of a combined M1 and H89 treatment *in vitro*, we next sought to explore the antitumor efficacy of this combination treatment in a subcutaneous xenograft model. Therefore, we developed subcutaneous tumor bearing nude mice with HCT-116 and Capan-1 cancer cell lines, which are refractory to M1 treatment alone. We intraperitoneally injected with H89 and intravenously injected with M1 virus. Compared with control group, either single treatment does not inhibit tumor growth. However, H89/M1 combination treatment group significantly inhibits tumor growth compared with the other three groups (Figure 6A and 6B). The schedule of the treatment was shown in Figure 6C.

H89 increases the oncolytic efficacy of M1 via inducing irreversible ER stress induced cell apoptosis. We next sought to investigate the tumor tissues with Immunohistochemistry (IHC) staining. With Ki-67 staining, which indicates the proliferation properties of cancer, we found that H89/M1 combination significantly downregulates the protein expression in HCT-116 and Capan-1 tumor mass (Figure 6D and 6E). With cleaved Caspase-3 staining, we observed that H89/M1 combination treatment induces cleaved Caspase-3 expression in HCT-116 and Capan-1 tumor mass (Figure 6D and 6E). These data suggests that M1/H89 combination treatment inhibits tumor growth and induces apoptosis *in vivo*.

## DISCUSSION

OVs are advancing through late-phase randomized clinical trials, and it seems likely that more agents will be approved after T-Vec [36–40]. Despite encouraging clinical advances, the combination of small molecule and oncolytic virus will lead to improved therapeutic outcomes. In this study, we show that, a classical PKA inhibitor, H89 selectively enhances oncolysis of M1 via activation of Epac1. Innate antiviral response is a critical barrier for oncolytic virus replication and spread within malignancy. Here, we find that H89 inhibits NF-κB transcriptional activities, thereafter, leading to induced antiviral response inhibition.

With a microarray analysis, we find that H89 inhibits diverse range of interferon stimulated genes (ISGs) expression induced by M1 through inhibition of p65. There are two possibilities that how Epac1 activation inhibits p65 nuclear translocation. (i) The rate-limiting step in the activation of NF-κB pathway in response to stimulation is the degradation of the IκB proteins, which inhibit NF-κB function by preventing the p65 nuclear translocation [41, 42]. Thus Epac1 activation might block this classical NF-κB pathway via stabilization of IκB. (ii) It is reported that cAMP-mediated suppression of p65 transcriptional activation is impaired in c-Fos deficient cells. c-Fos physically interacts with p65 protein and reduces the transcriptional activity of p65 [23]. Thus, Epac1 activation might upregulate c-Fos to inhibit nuclear translocation of p65.

H89 inhibits inflammatory response has been extensively reported [43, 44], however, the underlying mechanism that H89 activates Epac1 GDP-GTP exchange activities remains unknown. H89 is a multi-target molecule that regulates lots of targets and pathways [45–47]. Thus, on one hand, these pathways might indirectly modulate the Epac1 activities. On the other hand, we doubt that more cAMP might bind to Epac1 after PKA activity is blocked with H89.

Overall, our data shows that M1/H89 combination treatment dramatically inhibits cancer cell growth *in vitro*. The direct cell lytic effect is through prolonged and severe ER stress mediated apoptosis. Mechanistically, the enhanced oncolytic effect of M1 is through a previously unappreciated role of H89. Moreover, M1/H89 combination treatment inhibits tumor growth *in vivo* with systemic administration. At the same time, the combination treatment modality does not trigger death in normal cells and visible toxicity in mice. Notably, our data demonstrate that Epac1 pathway activation can provide a significant therapeutic benefit when combined with oncolytic virus M1.

## MATERIALS AND METHODS

### Reagents and cell lines

Cell lines (HCT-116, Capan-1, PLC, HCT-8, SW480, Hep3B and L02) were purchased from American Type Culture Collection and Shanghai Institute of Cell Biology. Primary cell lines (HH) were purchased from Sciencell Research Laboratories. All cell lines were maintained at 37°C with 5% CO_2_, in Dulbecco's modified Eagle's medium, supplemented with 10% foetal bovine serum (FBS), penicillin/streptomycin. Reagents used in this study are listed as follows: H89 2HCl (10 mm, dissolved in DMSO, S1582, Selleck, Houston, TX), dbcAMP (100 mm, dissolved in ddH_2_O, D0627-1G, Sigma, St. Louis, MO), Forskolin (20 mM, dissolved in DMSO, F6886-10MG, Sigma), ESI-09 (10 mM, dissolved in DMSO, B 133, Biolog, Germany).

### M1 viruses

Viruses were propagated in Vero cells (OPTI-SFM, 12309-019, Thermo Fisher, Waltham, MA). Virus titers were determined by TCID50 in the BHK-21 cell line and converted to PFU.

### Cell viability assays

Cells were seeded in 96-well plates at 4,000 cells per well, and were infected with M1 virus (10 PFU/cell) and various drugs were added as described in the figure legends where applicable. 72 hours later, cell viability was determined by MTT assays. Cells were stained with 3-(4,5-dimethylthiazol-2-yl)-2,5-diphenyltetrazolium bromide (MTT) at a concentration of 1 mg/ml. Plates were then cultured at 37°C for another 4 hours. Media was carefully removed and precipitates were then dissolved in 100 μl DMSO. The optical absorbance was determined at 570 nm using an iMark microplate reader (Bio-Rad).

### DAUC calculation

Dose response of cancer cells in the presence (b) or absence (a) of H89 were performed 72 hours post M1 virus infection. Areas under the curve (AUC) were calculated with Graphpad Prism 6 (La Jolla, CA). Difference in AUC indicates (area (a)-area (b))/area (b).

### RNA interference

Specific and scramble siRNAs were synthesized by Ribobio (Guangzhou, China). Cells were replaced with 10% foetal bovine serum (FBS) DMEM before transfection (without penicillin/streptomycin). siRNAs were then transfected using Lipofectamine RNAiMAX (13778-150, Thermo Fisher) with OPTI-MEM (31985070, Thermo Fisher).

### Antibodies and western blot analyses

Cells were lysed using M-PER Mammalian Protein Extraction Reagent (Thermo Scientific) and SDS-PAGE was performed. Antibodies used in this study are listed as follows: Human, CREB (9197, Cell Signaling Technology, Danvers, MA), phosphorylated CREB (9198, Cell Signaling Technology), JNK (9252, Cell Signaling Technology), phosphorylated JNK (9255, Cell Signaling Technology), Caspase-12 (2202, Cell Signaling Technology), GAPDH (AP0060, Bioworld, St. Louis Park, MN), PKAcα (4782s, Cell Signaling Technology), Ki-67 (9449s, Cell Signaling Technology), Cleaved-Caspase-3 (9664s, Cell Signaling Technology), p65 (8242, Cell Signaling Technology), phosphorylated p65 (3033, Cell Signaling Technology), M1 E1 and NS3 (produced by Beijing Protein Innovation).

### Transmission electron microscopy

HCT-116 cells were infected with M1 (10 PFU/cell) in the presence or absence of H89 for 48 hours. In brief, cells were scraped and pelleted at 1,000×g for 5 min at room temperature. Cell pellets were resuspended, washed once with PBS, further pelleted at 1,500×g for 5 min, and fixed for 4 hours in 0.1 M PBS (pH 7.4) containing 2.5% glutaraldehyde and 2% PFA on ice. Samples were then submitted to the Zhongshan School of Medicine (Sun Yat-sen University) Electron Microscopy Facility for standard transmission electron microscopy ultrastructural observation.

### Caspase activity analyses

Cells were cultured in 96-well plates and infected with M1 virus (10 PFU/cell) in the presence or absence of H89. Caspase-3, Caspase-9 and Caspase-8 activities were determined by Caspase-Glo Assay Systems (Promega, Madison, WI) according to manufacturer's protocols. The results were normalized to cellular viability (MTT assay).

### NF-κB transcriptional activity

Five thousand HCT-116 cells per well were seeded in 96-well plates and were allowed to settle for 12 hours. 100 nanograms of pNF-κB-luciferase plasmid or control-luciferase plasmid plus 10 ng pRL-TK renilla plasmid (Promega) were transfected into cancer cells by using the Lipofectamine 3000 reagent (Thermo Scientific). Medium was replaced after 12 hours, and luciferase and renilla signals were measured 12 hours after different treatment by using the Dual Luciferase Reporter Assay Kit (Promega) according to a protocol provided by the manufacturer.

### Microarray assay

Total RNA was extracted using TRIzol (Thermo Fisher) reagent and Samples were sending to CapitalBio Technology (Beijing, China) for GeneChip Human Genome U133 Plus 2.0 Array (Affymetrix). Microarray analysis was performed on biological duplicate samples.

### Immunofluorescent staining

Cells seeded in 6-well plate were exposed to different treatments and then washed, fixed, and permeabilized with 0.5% (v/v) Triton X-100/PBS and lastly blocked with 5% (v/v) BSA/PBS for 10 minutes. Primary antibodies (rabbit monoclonal anti-p65 antibody, 1 : 500 in 0.1% Triton X-100) and secondary antibodies (FITC-conjugated goat anti-rabbit IgG, 1:150 in PBS) were used to examine p65. Pictures were imaged by fluorescence microscopy (Olympus, Tokyo, Japan).

### Mitochondrial membrane potential assay

Fluorescent probe (5 μM), 5,5′,6,6′-Tetrachloro-1,1′,3,3′-tetraethyl-imidacarbocyanine iodide (JC-1, Sigma-Aldrich, St. Louis, MO), was treated on cultured cells seeded onto 6-well plate, and incubated for 20 minutes at 37°C. Cells were then washed with DMEM and photos were captured by fluorescence microscopy (Olympus, Tokyo, Japan) using a “dual-bandpass” filter.

### Animal models

Mouse studies were approved by the Animal Ethical and Welfare Committee of Sun Yat-sen University. HCT-116 (5×10^6^ cells/mouse) and Capan-1 (3×10^6^ cells/mouse) cancer cells were inoculated subcutaneously into the hind-flank of 4-week-old female BALB/c-nu/nu mice. After 5-7 days, palpable tumors developed (50 mm^3^), and mice were divided into 4 groups by random. The four groups were intravenously injected with OPTI-SFM or M1 (3×10^7^PFU/d) in a total volume of 200 μL. The four groups were intraperitoneal injected with DMSO or H89 (10 mg/kg/d) in a total volume of 50μL. Tumor lengths and widths were measured every other day and the volume was calculated according to the formula (length×width^2^)/2. Measurements were performed blinded to group allocations.

### Immunohistochemistry assay

The expressions of Cleaved-Caspase 3 and Ki-67 in tumors were characterized by immunohistochemistry using specific antibodies. In brief, tumor sections (4 μm) were dewaxed in xylene, dehydrated in descending concentrations of ethanol, immersed in 0.3% H_2_O_2_-methanol for 30 minutes, washed with PBS, and probed with monoclonal anti-Cleaved-Caspase 3 (1:100) or Ki-67 antibodies (1:100) or isotype control at 4°C overnight. After washing, the sections were incubated with biotinylated goat anti-rabbit or anti-mouse IgG at room temperature for 2 hours. Immunostaining was visualized with streptavidin/peroxidase complex and diaminobenzidine, and sections were then counterstained with hematoxylin.

### Epac1 activity assay

Cells were cultured in 55 mm plate and treated with Forskolin or H89 for 24 hours. Epac1 activities were determined by Active Rap1 Detection Kit (Cell Signaling Technology) according to manufacturer's protocols. Samples were then determined by western blot.

### Statistical analysis

All statistical analyses were performed using SPSS 13.0 software. Most of the data were analyzed by Student's t-test or one-way *ANOVA* followed by Dunnett's multiple post-hoc tests. Values of tumor volume were subjected to repeated measures *ANOVA.* Unless otherwise indicated, all error bars indicate Standard Deviation (S.D.). Significance was defined as P< 0.05.

### Study approval

All animal studies were approved by the Sun Yat-sen University Institutional Animal Care and Use Committee.
